# Breaking Barriers in Emerging Biomedical Applications

**DOI:** 10.3390/e24020226

**Published:** 2022-01-31

**Authors:** Konstantinos Katzis, Lazar Berbakov, Gordana Gardašević, Olivera Šveljo

**Affiliations:** 1 Department of Computer Science and Engineering, European University Cyprus, Nicosia 2404, Cyprus; k.katzis@euc.ac.cy; 2 Institute Mihajlo Pupin, University of Belgrade, 11060 Belgrade, Serbia; 3 Faculty of Electrical Engineering, University of Banja Luka, 78000 Banja Luka, Bosnia and Herzegovina; gordana.gardasevic@etf.unibl.org; 4 Faculty of Technical Sciences, University of Novi Sad, 21000 Novi Sad, Serbia; olivera.sveljo@uns.ac.rs

**Keywords:** biomedical, smart healthcare, Internet of Things, image compression, entropy, emerging technologies, applications, security

## Abstract

The recent global COVID-19 pandemic has revealed that the current healthcare system in modern society can hardly cope with the increased number of patients. Part of the load can be alleviated by incorporating smart healthcare infrastructure in the current system to enable patient’s remote monitoring and personalized treatment. Technological advances in communications and sensing devices have enabled the development of new, portable, and more power-efficient biomedical sensors, as well as innovative healthcare applications. Nevertheless, such applications require reliable, resilient, and secure networks. This paper aims to identify the communication requirements for mass deployment of such smart healthcare sensors by providing the overview of underlying Internet of Things (IoT) technologies. Moreover, it highlights the importance of information theory in understanding the limits and barriers in this emerging field. With this motivation, the paper indicates how data compression and entropy used in security algorithms may pave the way towards mass deployment of such IoT healthcare devices. Future medical practices and paradigms are also discussed.

## 1. Introduction

Technological advancements in the production of microelectronic components and wireless communications have enabled a myriad of new applications such as smart energy control, industrial IoT, smart agriculture, smart health, etc. One of the areas that has been shown to offer huge promises is smart healthcare. Initially, the focus was put on fitness tracking devices such as smart watches. Since then, new types of non-invasive miniature physiological sensors emerged, such as the electrocardiogram (ECG), oxygen saturation, blood pressure, etc. By building upon the initial momentum of fitness applications, these new devices allowed the wider usage of smart health applications in the general population. The recent coronavirus pandemic has proven that the world was not prepared for such a disastrous event. It has shown that remote healthcare applications are needed more than ever, especially in situations where hospitals cannot admit an increasing number of patients where many were treated in a home environment or temporary hospital. Due to unpredictable illness evolution, it is necessary to always have timely information about a patient’s vital signs, such as blood oxygen saturation, to be able to react in time and prevent illness progression.

In recent years, an increase in the aging population has been observed in many countries. Although this trend was initially observed mainly in developed countries, now it can be seen in many developing countries as well. It is predicted that, by 2050, one-sixth of the world’s population will be older than 65 years, in comparison to one-tenth in 2019. There is no doubt that these trends will contribute to a significant increase in the number of people needing support due to their disabilities and chronic diseases. Many of these patients can be successfully treated at home environment, thus allowing elderly and disabled people to have better life quality and at the same time the burden on the healthcare system is reduced. Nevertheless, this approach, known as Ambient Assisted Living (AAL) [1], requires the deployment of smart devices in the patient’s home and on the patient themselves, to monitor the patient´s state and react in time in case of deterioration of vital parameters. One of the main prerequisites to enable this new way of providing remote healthcare is to have reliable communication between the smart sensors and the healthcare provider. The adoption of novel and advanced technologies in the smart healthcare sector will improve the performances in terms of latency, interoperability and scalability, energy efficiency, security and privacy, etc. The design of communication protocols shall take into account the biomedical signals as well as the communication medium properties, as it is known from the Mathematical Theory of Communication [2] by C.E. Shannon.

In this paper, we elaborate on different aspects of biomedical signal communications from an information theory perspective. We contribute under the following perspectives: (1) by providing an overview of the state-of-the-art and emerging biomedical applications, and their communication requirements; (2) by reviewing different communication technologies and standards commonly used in the medical application; (3) by highlighting the benefits of advanced concepts such as Machine Learning (ML), Big Data Analytics (BDA), Software Defined Networking (SDN), biometric technologies, Blockchain, and Cloud/Edge/Fog computing, as foundations of IoT for biomedical and healthcare domain.

## 2. Emerging Biomedical Applications

With the aging population numbers rising, the sort of diseases that our current and future societies deal with are mainly cancer, cardiovascular disease, Parkinson’s, asthma, obesity, diabetes, and various other chronic or fatal diseases. In many cases, this sort of disease could be managed or even prevented if detected and monitored at an early stage. It is therefore imperative for aging societies to invest in a future healthcare system that will be able to cope with a large aging population. The future healthcare system should be in a position to integrate human medical expertise and services along with modern technology to provide proactive wellness management and concentrate on the early detection and prevention of diseases. Such medical-oriented technologies have been evolving for some time, driven by the needs of society and of the general market. According to [3], even monitoring vital signs can be considered as a key solution for offering proactive healthcare services and this can be achieved through a network consisting of smart, low-power sensors and actuators. Such devices may be used on-body (wearable) or in-body (implanted), or even in the blood stream providing regular and accurate data. As with many products in our lives, it is natural that such solutions are market-driven and based on facts and figures worldwide dictating which type of diseases affect our population.

**Figure 1:**
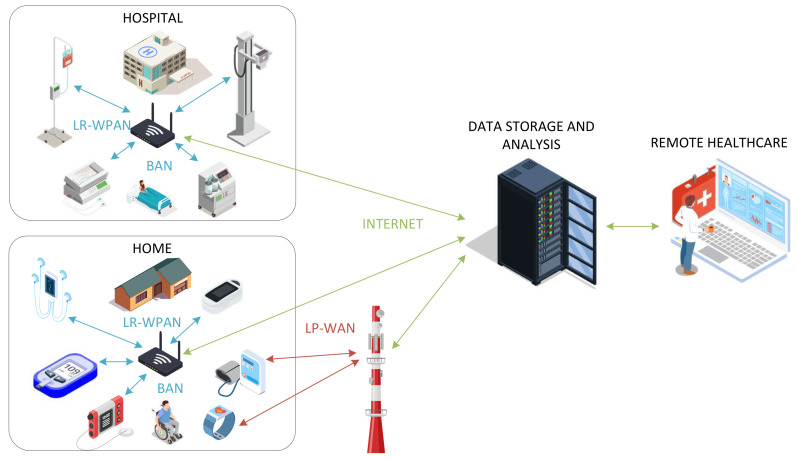
Remote healthcare system.

Figure 1 provides an overview of the remote healthcare system, where both hospital and home environment are considered. Furthermore, it presents different wireless communication networks, which will be discussed in subsequent sections and are used to connect equipment and sensors used to monitor the patient’s vital signs. The measurements which are collected at the gateways and base stations are conveyed over the Internet to the server where the data are stored and processed. Finally, these data are presented to the end-user: healthcare practitioner or the patient via the web, desktop, or mobile applications.

### 2.1. Use Cases

In order to define what kind of technologies are expected to deliver medical sensing data to support future healthcare services, it is necessary to first look at the use cases that have been identified. These use cases have been selected based on their operation, transmission characteristics, and market penetration:

#### 2.1.1. In-Body Sensors

- Glucose Sensors (GS): These are widely used across the globe. They are used to measure the blood glucose concentration and help patients cope with diabetes mellitus. In recent years a new type of GS has been developed. Such sensors are typically implanted under the skin [4], featuring an interface circuit while they offer continuous measurement and monitoring of blood glucose in patients with diabetes. A GS will typically transmit at a rate below 1 kbps with expected latency under 150 ms. According to the World Health Organization, the number of people with diabetes rose from 108 million (approximately 2.4% of the entire population) in 1980 to 422 million (approximately 5.8% of the entire population) in 2014, while in 2019 diabetes was the ninth leading cause of death with an estimated 1.5 million deaths directly caused by diabetes [5]. It is worth noting that diabetes is found to be significantly more common in urban than rural areas [6,7,8]. This means that in urban areas, the network must be able to cope with a high density of users, causing significant traffic to the network.
- Pacemaker: Cardiovascular diseases are among the most common diseases across the world. Pacemakers have been invented to sense irregularities in heart beating and to send a signal to the heart that makes it beat at the correct pace. A pacemaker is a generally small, battery-operated device. The typical data rate expected to be transmitted is below 1 kbps for a 12-bit pacemaker with a 500 Hz sampling rate. The latency expected for a pacemaker should be less than 150 ms. According to [8], cardiovascular diseases are the leading cause of death globally, taking an estimated 17.9 million lives each year. According to [9], rural residents experience higher death rates compared with residents of urban areas. In many cases, the cause of this death could have been prevented [10]. A pacemaker along with an ECG can prove lifesaving, offering vital information about the state of the heart. As with the glucose sensor, it is expected that IoT networks in urban areas will be required to support this critical data in real-time, while in some cases priority will be given to people in critical conditions. At the same time, it is expected that this network will have to handle the traffic found in urban densely populated areas.
- Capsule Endoscopy (CE): This has been possible thanks to modern miniaturized electronics and Ultra-Wideband (UWB) communications that enable the investigation of the small bowel providing a non-invasive, well-tolerated means of accurately visualizing the distal duodenum, jejunum, and ileum [11]. CE is typically used when flexible endoscopy fails to identify a diagnosis. It consists of a capsule for imaging, a device for receiving and storing data, a computer, and software. The capsule itself consists of a light source, lens, battery, and a transmission device. A CE would typically require 1 Mbps of data rate with less than 150 ms of latency.

#### 2.1.2. On-Body Sensors

- Electromyography (EMG): This measures the electrical activity of muscles by recording the electrical impulses that muscles produce. The nerve conduction test measures the speed at which impulses travel along a nerve during rest, slight contraction, and forceful contraction. A 12-channel EMG requires 1.536 Mbps of data rate at 8 kHz of sampling rate. The latency should be less than 250 ms. This implies that the communication channel carrying this sort of data will have to have enough bandwidth to meet these transmission requirements. Such application can be used to examine changes in muscle activity during acute and subacute phases of stroke recovery [12].
- ECG: As mentioned before, cardiovascular diseases are among the most common and life-threatening diseases across the world. People who suffer from cardiovascular diseases are usually fitted with an ECG, which measures changes in electrical signals on different areas of skin. Such signals are basically electrical and chemical signals used to enable communication between our nerve and muscle cells. Regular electrical signals also control our heartbeat. An ECG is mainly used for recording how often the heart beats (heart rate) and how regularly it beats (heart rhythm). In some cases, during the monitoring process, patients are encouraged to continue their daily routines in order to be able to capture possible irregularities of the heartbeat. A 12-channel ECG requires 72 kbps data rate to communicate recorded data at a sampling rate of 500 Hz and latency of less than 250 ms. As with the EMG, the communication channel required for carrying this sort of data need to have enough bandwidth to meet the transmission requirements.
- Temperature, Heart Rate (HR), Blood Pressure (BP), Blood Oxygen (BO): The main vital signs of the human body such as Temperature, HR, BP, and BO are associated with a single value that is not expected to change rapidly within a given time. Thus, the data rates required for such applications are expected to be below 10 kbps employing low power channels for the data transmission. Vital signs monitoring provides an easy way for the early detection of various diseases. The subtle variation of vital signs, such as the core body temperature, can be a significant indicator for older patients as it often indicates a more severe infection and is associated with increased rates of life-threatening consequences [13].

#### 2.1.3. Intelligent Things in Smart Hospitals

- Automated Medicine Dispenser: Automated dispensing machines are used to securely store medication on patient care units. They also feature electronic tracking of the use of narcotics and other controlled drugs. These machines can save nursing time by eliminating a need for manual end-of-shift narcotic counts in the patient care units. Another clinical feature of automated dispensing machines is the capability to track and proactively monitor drug usage patterns. This is accomplished by setting up clinical indicators during the removal of specified drugs [14].
- Urine Monitoring System: According to [15], a urine monitoring device has been designed and implemented for monitoring postoperative urination. This device has been designed to reduce the burden of the nursing staff required to regularly monitor and empty the urine bags as well as to provide crucial information about the rate of flow of urine in real time that is vital information for postoperative urination. Authors have implemented this using WiFi, but this assumes adequate WiFi coverage across the hospital. Using Low Power Wide Area Network (LP-WAN) type of technology should alleviate this burden and enable the mass deployment of such low-cost devices in a hospitalized environment.
- Intensive Care Unit (ICU): An ICU is a true example where information technology and clinical informatics are used to acquire, process, and transform data into actionable information. Furthermore, it is required that this information disseminates effectively to improve patient care. Intensive care in ICUs involves highly complex decision making based on data [16]. In general, this is real-time data and the whole decision-making mechanism must provide decisions in real time. The basic approach of collecting and managing the data involves communicating data from many disparate sensors into a database/system (possibly located at the hospital). The system must be reliable, resilient, and responsive to rapid changes recorded by the sensors. For the ICU scenario, we consider that some of these devices can be connected using the IoT network available at the hospital, especially in emergency erected ICUs such as the one’s setup during the pandemic [17]. Devices (sensors/actuators) used in an ICU are automated medicine dispenser, the infusion monitoring system, ECG, vital signs, respiratory ventilator, syringe pump, infusion pump, etc.
  According to [18], to connect intelligent things using Narrow Band Internet of Things (NB-IoT) architecture, there are still numerous challenges to be addressed. Such challenges are the limited accuracy and reliability of data collection, which is a major challenge in the building of smart hospitals. Furthermore, security is a general challenge since the IoT network will have to handle sensitive/critical data. The encryption mechanism of terminal devices must employ an encryption algorithm and key management mechanism to strengthen authentication.

### 2.2. Communication Requirements and Network Limitations

Based on the use cases presented above, such medical devices operating within a smart environment must transmit their data to a safe location where it will be processed. Each medical device can be considered as a source of generated data. In real life, however, patients or even healthy people in general may require a combination of these sensors in order to monitor a particular medical condition. Thus, it is important that the communication requirements be clearly set to define the network that will drive these use cases in large numbers while combing various scenarios together.

According to [19], the estimated growth of the IoT through connected devices across the globe will increase from 7.6 billion at the end of 2019 to 24.1 billion in 2030. This was translated into significant revenue, increasing from USD 465 billion to over USD 1.5 trillion by the year 2030. It is expected that some of the IoT applications will require low-rate, long-range, and delay-tolerant wireless communication at very low energy usage and cost. According to [20], the market trend indicates that the number of mobile phones has saturated since users do not require more than 1–2 mobile devices. However, this is not the case with the “things” since their numbers are increasing exponentially while their data traffic pattern significantly differs from the general human-centric data traffic. We can consider a scenario where a number of such medical devices, measuring vital signs, operate within a given geographical area. In most cases, devices are in sleep or idle mode most of the time, saving their precious battery resources. Nevertheless, according to the Emergency Safety Index (ESI)-3, patients with normal vital signs should be reassessed at the discretion of the nurse, but no less frequently than every 4 h (i.e., six times a day). So, it is expected that these signs should be measured and transmitted through the IoT network at least six times a day. Considering the city of Paris as an example, Paris has a population density of 20,909 per square kilometer. Assuming that about 1% of the population may require daily monitoring of their vital signs, glucose levels, and heart monitoring using an ECG, this accounts to a minimum of 200 people per square kilometer. Table 1 lists all these sensors and presents the frequency of their transmission and the size of the data generated every time they communicate with the IoT network. All these data must be managed by the IoT network on top of the existing data generated by all other IoT devices that people are using.

According to [20] and based on nine basic use cases, it is expected that 7800 devices will be operational in one service area of 1000 households. Compared to our reference city of Paris, for a population of 20,909 people per square kilometer and with the number of persons per household set at 2.38 [26], this translates to about 68,525 devices per square kilometer. We can therefore safely assume that a large number of devices will need to be managed by the IoT networks available in a given area; hence, there is an urgency of planning and rapid deployment of a number of LP-WAN networks in cities to address the different types of traffic requirements generated from a wide range of IoT-enabled devices, both medical and non-medical.

## 3. Communication Standards in Medical Applications

Advancements in communication technologies represent one of the main pillars that enable wider usage of biomedical applications. Although many of the already existing communication protocols can be successfully applied in biomedicine, there still exists room for improvement of their performance. In this section, we provide a review of different communication protocols which can be grouped into two categories:

- Low Rate Wireless Personal Area Network (LR-WPAN);
- Low Power Wide Area Network (LP-WAN).

### 3.1. LR-WPAN

LR-WPAN communication protocols are used in scenarios characterized with low data rate requirements, where the focus is more on the higher energy efficiency than the data throughput. The application of IoT sensor devices in smart healthcare is aimed at providing reliable transfer of measurements such as blood pressure, pulse, blood glucose level, ECG, etc. [27,28,29]. These data are usually collected at a gateway concentrator and sent to a remote server where they are usually processed by different data analysis algorithms, such as machine learning. There already exist a number of Wireless Personal Area Network (WPAN) protocols that can be applied in biomedical applications, such as Bluetooth Low Energy (BLE) [30], ZigBee [31,32], and NFC [33], which are usually used to transmit the data to the gateway concentrator. LR-WPAN networks are suitable for collection of measurements from in-body sensors, such as the glucose sensor [34], pacemaker [35] and endoscope capsules [36], due to the lower signal ranges and requirement for energy efficient operation [37].

BLE, which is also called Bluetooth Smart, was designed in 2006 and included in the main Bluetooth standard as a part of Bluetooth Core Specification version 4. So far, it has been mainly used in consumer electronic devices such as smart watches, tablets, mobile phones, and different home IoT devices such as sensors, lamps, etc. In contrast to the original Bluetooth standard, which is optimized for continuous data transmission aimed at file transfer and music streaming, BLE is aimed at transmission of short messages. Newer Bluetooth standards such as Bluetooth 5.0 and 5.1 provide additional features such as higher data rate and enhanced interoperability, which make them even more suitable for smart healthcare applications.

The ZigBee protocol has mainly been used in smart home applications. It has been developed to become an open communication standard aimed at responding to the requirements of high energy efficiency for Machine to Machine (M2M) communication. It is based on the IEEE 802.15.4 standard, which works in unlicensed frequency bands including 868 MHz, 900 MHz, and 2.4 GHz, where the selection of band depends on local wireless transmission regulations. This standard is built in a way to allow devices to communicate in different network topologies such as point-to-point, mesh, and star. The ZigBee protocol has high energy efficiency, which allows it to work for years from single batteries. Since it uses unlicensed bands, it needs to employ interference avoidance techniques in order to cope with the interference from other devices that share the same frequency band, such as Bluetooth and WiFi. Maximum transmission range is limited to 10–150 m, depending on the propagation environment and transmitter output power. In order to deal with this limitation, mesh networking mechanisms are used. Namely, by using intermediate network nodes—Zigbee routers—the data are relayed through a series of hops. Zigbee is commonly used in applications requiring low data throughput up to 250 kbps and higher energy efficiency and security (128 bit Advanced Encryption Standard (AES) data encryption). It is built upon the IEEE 802.15.4 standard where network and application layers are further specified by the Zigbee protocol.

### 3.2. LP-WAN

LP-WAN networks are used to enable the long range transmission with low transmit power and higher energy efficiency. This technology is especially useful when there is a need to provide long battery lifetime of the sensing devices such as thermometer [38], HR meter, and oxymeter [39] that send the measurements with lower data rate over longer ranges. LP-WAN networks that operate in licensed bands have the advantage of the dedicated spectrum which lead to lower interference levels and more reliable communications, which is particularly suitable for critical healthcare applications.

- NB-IoT is envisioned to support Wireless Sensor Networks (WSN) within legacy cellular networks. Over time, some of the older generations of cell networks have become outdated. NB-IoT is expected to further increase their life time and bring new business models with a simple software update of the network infrastructure. These networks have narrow bandwidth of only 180 kHz which can be assigned within LTE guard band. Although such narrow bandwidth supports lower data rates, at the same time they provide extended coverage and reduced power consumption [40].
- Long Term Evolution-Machine Type Communications (LTE CAT-M) [41] enables connectivity of IoT devices with reduced complexity of the device. This technology can be implemented on the already existing LTE base stations. Nevertheless, it supports longer communication range and longer battery lifetime. Besides being based on the currently available mobile networks, LTE CAT-M has enhanced security and privacy, which is especially important when dealing with patient’s health data. It uses 1.4 MHz bandwidth in contrast to regular LTE which used 20 MHz, and supports the data transfer speeds of up to 1 Mbps, which is particularly suitable for healthcare monitoring that need higher data rates.
- Extended Coverage GSM Internet of Things (EC-GSM-IoT) represents the standard that operates within GSM frequency bands which is based on eGPRS [42]. It is capable of providing extended range with higher energy efficiency for IoT applications. Since this technology is based on the legacy GSM networks, their lifetime can be effectively extended and bring new business opportunities to the mobile network operators. It provides maximum data throughput of 240 kbps while requiring 200 kHz of bandwidth. Similarly to NB-IoT technology, this is suitable for healthcare applications requiring lower data rates. The battery life of connected devices is expected to be up to 10 years.

In addition to the aforementioned technologies, there also exist LP-WAN technologies which operate in unlicensed bands. These can be used in healthcare application which are not of critical importance, since the IoT devices need to share the available spectrum with other devices that also operate in Scientific and Medical (ISM) frequency bands.

- Long Range Wide Area Network (LoRaWAN) operates in sub-GHz frequency band and employs a proprietary spread spectrum modulation technique. It has been designed with the aim to support the mobile and fixed devices that are powered with batteries, where the energy efficiency is one of the most important aspects. In contrast to ZigBee, LoRaWAN is based on a star topology where different gateways communicate with the network nodes. It envisions three device types, based on the way they communicate with the network. More specifically, Class A enables bidirectional communication between network node and a gateway. For the uplink transmission, the data is randomly transmitted, whereas for the downlink, the receiver is turned on 1 and 2 s after the uplink transmission. Class B works in a similar manner where the receiving window is scheduled, while Class C enables bi-directional communication with low latency by allowing the receiving windows to be open at any time. LoRaWAN allows data rates up to 37.5 kbps [43] and transmission range of about 30 km, where these depend on the transmitter configuration, such as Tx power, signal bandwidth, spreading factor and propagation channel characteristics [44].
- SigFox represents a communication technology based on Shift Keying (DBPSK) and Gaussian Frequency Shift Keying (GFSK). It uses only 100 Hz out of total 192 kHz of total spectrum. Besides this, Sigfox message payload is limited to 12 bytes, with a limit of only 140 messages per day. In Europe, it operates on 868 MHz, whereas in North America a 902 MHz band is allocated for its use. The data received by the gateways are collected on the SigFox cloud servers and made available to the end users through an Application Programming Interface (API) or web-based interface. The limits of the number of messages per day and message size makes it suitable for non-critical scenarios with less frequent measurements [45].
- INGENU operates on ISM 2.4 GHz frequency band, which allows for wider usage since this band is less regulated worldwide. For the uplink communication, it employs Random Phase Multiple Access (RPMA) Direct Sequence Spread Spectrum (DSSS) as the transmission scheme. By using such an approach, multiple transmitters are able to share one time slot. Since each RPMA channel takes only 1 MHz of bandwidth, it is possible to have 40 uplink and downlink channels within 2.4 GHz spectrum. INGENU supports data rate of 19.5 kbps for downlink and 78 kbps for uplink. Transmission can reach up to 3 km in urban and up to 15 km in rural environments [46]. For security, it employs AES 256 bit encryption.
- WEIGHTLESS is a protocol that enables two-way communication by using PSK/GMSK and O-QPSK modulation with spread spectrum [47]. Similarly to other LP-WAN communication standards, it also uses sub-GHz frequency bands that do not require license. It supports adaptive bit rate from 625 bps to 100 kbps, while its channels are only 12.5 kHz narrow [48]. In order to optimize on the network capacity, WEIGHTLESS uses control of power in uplink and downlink directions. Finally, in order to enable secure transmission of data, it employs AES encryption with 128 and 256 bits.

## 4. Advanced Concepts in IoT for Biomedical Applications

Rapid prototyping and deployment of IoT devices have introduced an open space for the next generation applications in various fields such as industrial automation, smart cities, intelligent transportation systems, remote healthcare monitoring, etc. These applications generate large quantities of real-time data, thus providing significant benefits, but also introducing some critical challenges that should be carefully examined. In the emerging field of IoT for biomedical applications, ML, BDA, SDN, biometric technologies, Blockchain, and Cloud/Edge/Fog computing could be synergistically used to provide advanced networking architectures and solutions. During the last decade, the expansion of M2M communications has introduced some novel forms of interactions and data generation mechanisms with great potential for healthcare applications. In these interactions, large volumes of data are continuously being generated from various heterogeneous sources and transmitted for remote medical diagnostics, thus raising the challenges in data acquisition and analysis [49,50]. The scalability and interoperability of the IoT healthcare network infrastructure should be supported by providing Quality of Service (QoS) guaranties.

For scientists and researchers, the ability to collect and analyze large medical and healthcare datasets is an essential precondition in assuring timely disease detection, diagnosis, and treatment. The use of ML has already shown promising results in various biomedical and healthcare applications [51,52]. Among other ML techniques, supervised ML provides required support for time-critical healthcare applications. In combination with fog computing, it creates a powerful tool for robust end-to-end encryption [53]. Numerous techniques have been proposed for ECG signals analysis [54,55], where the most popular ECG databases are the MIT-BIH arrhythmia [56] and the AHA [57].

Entropy is one of the fundamental features of information and communication theory [2], widely used to study the complexity of biomedical signals [58,59], or the subtle interrelationships of multiple biomedical time series [60,61]. Biometric technology used in patient identification can significantly improve the speed and reliability of activities, as well as continuous authentication. It exploits physiological and behavioral biometric traits, including face, iris, fingerprints, ECG, and Photoplethysmography (PPG) [62]. The importance of entropy is confirmed in the healthcare domain since it can be used for the production and certification of IoT devices for biomedical applications with the high-security level [63]. One possible approach is presented in [64], where the noise-aware biometric quantization framework (NA-IOMBA) generates reliable and high entropy keys with low enrollment times and costs. Experiments are performed on four biometrics modalities (ECG, PPG, fingerprint and iris scan). The results show that NA-IOMBA outperforms them all and that ECG-based authentication provides the best trade-off between reliability, key length, entropy, and implementation cost.

The secure transmission of medical images is another challenging task since traditional encryption techniques cannot ensure the required level of privacy and confidentiality of patient data transmitted in heterogeneous IoT environment. Recent research efforts generate advanced image encryption techniques particularly suitable for medical images [65,66]. One possible approach is presented in [67]. The encryption technique uses three stages to encrypt the image by using the 256 bits key value for logical operation. This lightweight framework provides high performance in terms of encrypted entropy, small computational time, encryption speed, and low complexity.

Cloud/Edge/Fog computing and IoT networking have emerged as a new level of interaction between people and devices. In the field of IoT for biomedical applications, these technologies provide required resources for processing, storage, analytics, and networking of data. Various types of IoT and cloud computing services enable continuous monitoring, predictive and preventive care, AI-driven diagnosis, etc. Among other relevant topics, data security and patient privacy are of paramount importance [68]. Entropy-as-a-Service (EaaS) is a cloud-based platform for the generation and distribution of high-quality entropy for distributed applications and IoT devices, as well as embedded computer systems [69]. EaaS provides the entropy for generating strong cryptographic keys required in prevention of malicious attacks.

Cloud computing services provide on-demand storage and analysis, but may also cause unpredictable latency with a negative impact on QoS. Potential issues include privacy and reliability of patient data, heterogeneous and resource-constrained devices, and complex monitoring and management. Therefore, the cloud-based IoT platforms for biomedical applications should be further explored and examined.

The softwarized infrastructure for smart healthcare provides significant benefits in terms of resource management and dynamic reconfigurability [70]. SDN introduces a control plane that implements logical functionalities of the network and a data plane to manage the actual transmission of user data [71,72]. This is particularly important for providing interoperability between different communication protocols, the management of constrained resources, as well as the support for the critical flows in IoT healthcare networks. For example, SDN-based Edge computing can provide efficient resource utilization in the healthcare system [73]. This solution provides the authentication of IoT devices by the Edge servers using a lightweight authentication scheme. An SDN controller performs load balancing and network optimization.

Novel solutions in computing and networking, as well as in data analytics, open the path towards new treatments for diseases, and drastically improve diagnostic quality by providing superior decision support to healthcare professionals. Analytics in Fog computing is performed closer to the edge of applications and devices, and this approach is more appropriate for events requiring a timely response. The distributed solutions could provide optimal performances depending on the specific biomedical application needs. In [74], IoT-fog based healthcare monitoring system is proposed for continuous monitoring of BP and other health parameters to identify stages of hypertension. The artificial neural network is utilized for the prediction of the risk level of hypertension. The Fog system enables continuous generation of an alert of BP fluctuation on users’ mobile phones, while results of analysis and medical information of each user are stored permanently on cloud storage.

Efficient deployment of WSN/IoT biomedical applications requires advanced security mechanisms and algorithms. Blockchain addresses the privacy concerns for IoT networks by using cryptographic algorithms for the robustness against failure and data exposure [75]. In [76], blockchain technology is used for remote healthcare applications. The model provides reliable data communication over the network and storage over the Cloud-based on lightweight cryptographic techniques. The concept of Ring Signatures that provides privacy properties like Signers Anonymity and Signature Correctness, has been introduced.

Some recent research activities address the challenges in collecting, storing, and processing large-scale biomedical datasets across heterogeneous computing and storage platforms [77]. As a result, Personal Health Dashboard (PHD), a secure, scalable, and interoperable platform for the streamlined and cost-effective acquisition, storage, and analysis of large biomedical datasets ranging from wearable biosensor data and multi-omics profiles to clinical data, is presented. Omics data contains catalog of molecular profiles which creates the base for precision and personalized medicine [78,79]. PHD can operate on any large-scale cloud infrastructure or local high-performance computing system and can be customized according to end-user needs. Moreover, the secure cloud-agnostic platform allows data capture, integration of diverse datasets, analytics, visualization, and notification to patients via the mobile PHD smart application.

In [80], the Dynaswap cybersecurity architecture has been proposed. This architecture combines existing security frameworks such as the Authentication Authorization Accounting framework, Multi-Factor Authentication, Secure Digital Provenance, and Blockchain with advanced security tools (e.g., Biometric-Capsule, Cryptography-based Hierarchical Access Control, and Dual-level Key Management). This solution comprises two major components: decryption and ML clusters for data processing and biomedical research, respectively. The security technologies have been developed and integrated with the Open Medical Record System to provide advanced security and privacy protection, as well as the remote access and share of sensitive data.

The use of cryptanalysis for authentication purposes provides an efficient mechanism for the protection of possible security threats. A novel genetic algorithm for data security architecture that performs one-time key, single block enciphering is presented in [81]. The algorithm combines Gene Fusion and Horizontal Gene Transfer inspired by the spread of antibiotic resistance in bacteria. A novel scheme for highly random variable keys results in an avalanche effect with an average of 98%. The architectural framework of the biomedical Wireless Body Area Network (WBAN)/IoT system is presented, both with theoretical and simulation analysis, where experimental results on low-cost electronic device prototype are also provided. In Table 2, we provide the summary of requirements for different healthcare applications and communication technologies.

## 5. Compression as the Past and Future of Medical Information

The principles of the techniques described in the previous sections arose from one basis—information theory—and its main disciplines developed during the narrow time period from 1948 to 1953: cryptography [86], channel capacity [87], and coding [88]. However, emerging applications that bring the future to healthcare are also capable to produce data in quantities that will soon deserve to be called “big”.

A key component for transmitting and storing such vast amounts of data is another pillar of information theory: compression [2,87]. Compressed data is more compact, reduces transmission and storage costs, and allows signal reconstruction either accurately (lossless) or with some distortion.

### 5.1. Piling the Cardiovascular Data

The ECG is a signal of modest size, and seemingly, its compression is superfluous. However, according to the World Health Organization (WHO), cardiovascular diseases account for the majority of premature deaths from non-communicable diseases [89]. Simple and non-invasive recording and low-cost equipment make the ECG as one of the most common diagnostic tools. It is used permanently during systematic examinations, home visits, in patients with private electrocardiographs, in hospitals, and during constant 24 h monitoring (“Holter” devices). E-health, telemedicine, crowdsensing, and crowdsourcing are services that perform the monitoring remotely and transmit and store data [90]. A simple Holter system with three electrodes and a modest A/D conversion with 200 Hz and 10 bits/sample generates 0.5 Gbit per patient per day. So the signals are already piling up, and the applications envisaged in the previous sections tremendously increases their transmission and storage.

The elementary method for lossy ECG compression is at the core of information theory: digitalization. A comprehensive analysis of the requirements for ECG sampling frequency and amplitude resolution appeared in 1990 [91]. The study followed the recommendations of the American Heart Association, as well as earlier contributions on the relationship of amplitude resolution and sampling frequency. Three characteristic of ECG implementations are outlined: visual observation, computerized analysis, and transmission/storage. The conclusion was in favor of a sampling frequency of 250 Hz for visual observation, and 500 Hz for computerized analysis and storage, with not less than 10 bits/sample. The recommendations of Task Force of the European Society of Cardiology and the North American Society of Pacing and Electrophysiology [92], also state the optimal sampling range of 250–500 Hz. The range of 100–250 Hz may be satisfactory as well, but only if an interpolation algorithm is applied. It is in accordance with A/D conversion followed by the complete implementation of the sampling theorem [37,87].

**Figure 2:**
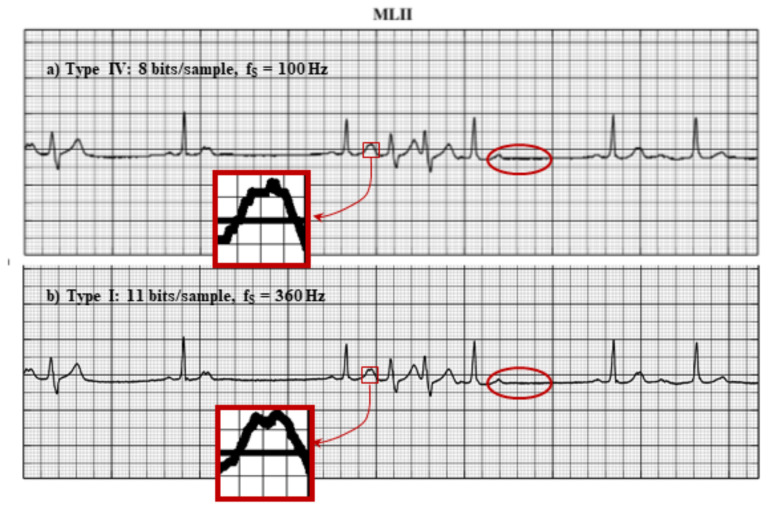
ECG signal from the MIT database. (**a**) Type IV—worst, $f_{s}$ = 100 Hz with 8 bits per sample; (**b**) Type I—best, $f_{s}$ = 360 Hz with 11 bits per sample. The rounded region in panel (**a**) was misinterpreted as a tremor; panel (**b**) shows that the alleged tremor is a consequence of low resolution.

In a 2002 experiment [93], cardiologists were asked to rank the quality of the recorded ECG groups of signals from the MIT database [94,95]. Each signal group consisted of the original record—360 Hz and 11 bps (ECG type I)—and the same record was processed to mimic 240 Hz, 10 bps (type II), 180 Hz, 9 bps (type III), and 180 Hz, 8 bps (type IV), Figure 2). Although the first signal produces five times more bits per second than the fourth, cardiologists equally ranked all of them, giving an insignificantly worse grade to the fourth.

**Figure 3:**
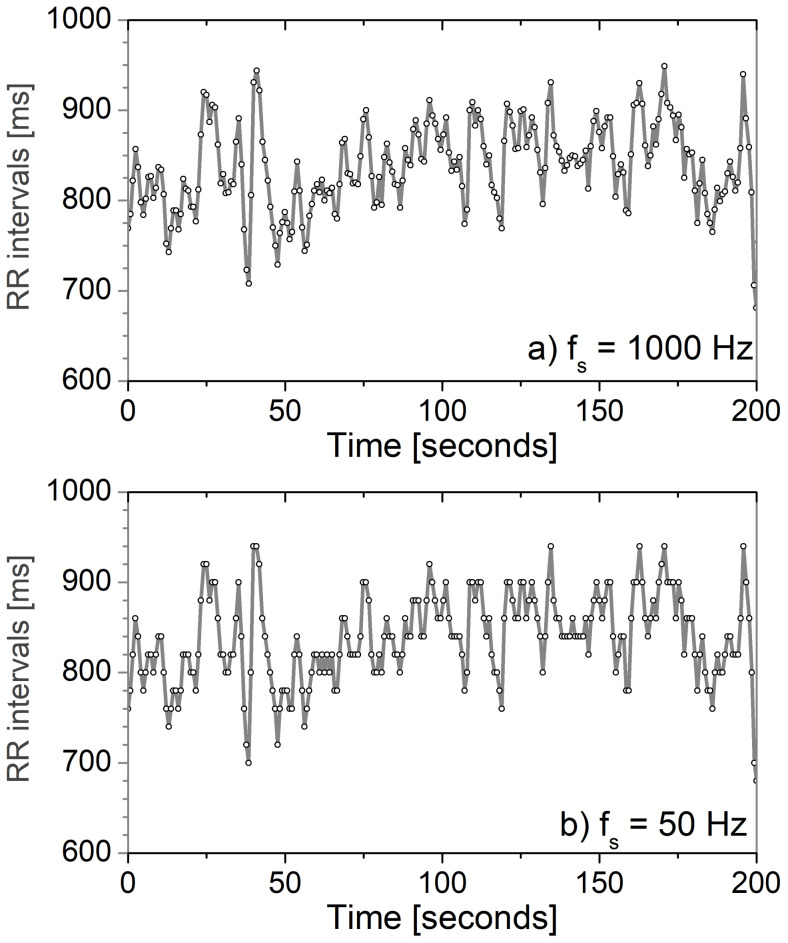
Time series of the RR interval of a healthy volunteer; (**a**) $f_{s}=1000$ Hz, with 200 different amplitude values within a range of 700 to 900 ms; (**b**) $f_{s}$ = 50 Hz, with only 10 amplitude values within the same range.

A recent study [96] relaxed the sampling frequency requirements, stating that 250 Hz is sufficient for any analysis, 100 Hz is acceptable for the time domain, but not for spectral analysis, while 50 Hz is not acceptable at all. Indeed, when the 1000 Hz signal is reduced to 50 Hz, the intervals between adjacent R peaks of the ECG signal get about 20 different values, so the corresponding time series of RR intervals becomes discrete (Figure 3). This violates the theoretical requirements for the analysis.

A “true” compression begins after digitalization. The state-of-the-art ECG compression methods from the nineties are reviewed in a classical paper [97]. The lack of standards considering the signal acquisition made the direct method comparison impossible, but the paper describes their advantages and disadvantages.

A more recent review [98] from 2015 partitioned the compression techniques into “time-domain”, “transform-domain”, and “2D”. An additional technique, “parameter-extraction”, is irreversible and does not provide signal reconstruction, so it does not fit the definition of data compression. The time-domain methods, mostly from the previous century, announced many techniques, among the most distinguished are “Aztec” [99] and “Cortes” [100]. More recently, novel techniques came into the focus, such as neural networks [101].

Considering the transform-domain, early techniques are summarized in [102], while recent ones are based on wavelet transforms [103,104,105]. The 2D techniques achieve better performances as they do not observe ECG sample by sample, but create an “image” of successive RR intervals [106].

The task of finding the proper compression method for a signal that seems so simple is still not finished. As already said, without the standardized acquisition procedure, the methods cannot be accurately compared. Subjective measures are important for visual ECG examination in a doctor’s office, but only objective measures enable direct comparison. The most common ones show compression performance and average signal distortion. Such measures are compression ratio—the ratio of the number of bits in original and in compressed data; and percent mean square difference—square root of the ratio of the sum of squared difference of the source and compressed signal amplitudes, divided by the sum of squared source amplitudes, expressed in [%]. However, the features of the signals—such as characteristics of QRS complex, and P and T waveforms, are gaining importance in novel diagnostic procedures. So the measures that estimate the preservation of signal features, rather than overall signal distortion, become more important. One of the most distinguished ones is weighted diagnostic distortion, which checks 18 parameters of the signal [107]. Despite its complexity, it checks the preservation of the most relevant features of the compressed ECG signal.

Since 2000, the widely used Digital Imaging and Communication in Medicine (DICOM) standard, originally developed for image storage, has included rules for diagnostic ECG. A few years later, the first ECG manufacturer adopted the DICOM standard for diagnostic electrocardiographs [108], followed almost immediately by appropriate PACS solutions [109]. In this way, proprietary systems are no longer required to exchange ECG data.

### 5.2. Medical Image Compression

The number of medical image examinations is constantly increasing. There are more and more imaging procedures and the number of images per examination is increasing, as well as the image resolution. For example, during 2019–2020, more than seven million Computed Tomography (CT) and Magnetic Resonance Imaging (MRI) examinations were performed in Canada, and the number of CT scans per thousand people was 143.4. According to the OECD (Organization for Economic Cooperation and Development), this puts Canada in the lower quarter among the observed countries [110].

The size of uncompressed medical imaging files from one patient could be from a few MB for nuclear medicine to 1 GB for advanced CT exams [111]. The healthcare procedures assume that all acquired images are securely stored and are available for secure and as fast as possible transmission on demand, which actualizes basic information theory problems on coding, transmission, and data compression [2] in medical imaging.

The problem of storage and transmission of medical images and the need for standardization has been recognized from the early 1980s with the introduction of MRI as the new intrinsically digital medical imaging modality (the first was CT in the early 1970s). The American College of Radiology (ACR) and National Electrical Manufacturers Association (NEMA) formed the Digital Imaging and Communication Standards Committee in 1983. The first version of the standard (ACR-NEMA 300-1985) was introduced in 1985 and the first revision was published in 1988. The standard has been continuously improved and the standardization committee has been expanded to manufacturers outside the United States and medical specialties beyond radiology. In 1993, the name DICOM was established. Today’s release of the standard specifies a medical image and patient data file format as well as the network communications protocol [112]. In NEMA nomenclature, DICOM is known as NEMA PS3, while in ISO standards, DICOM is denoted as ISO12052:2017: “Health informatics—DICOM including workflow and data management”. DICOM is widely accepted in the healthcare industry and there is a general opinion that DICOM standards have a crucial role in the transformation of film-based medical imaging to full digital workflow in modern hospitals.

**Figure 4:**
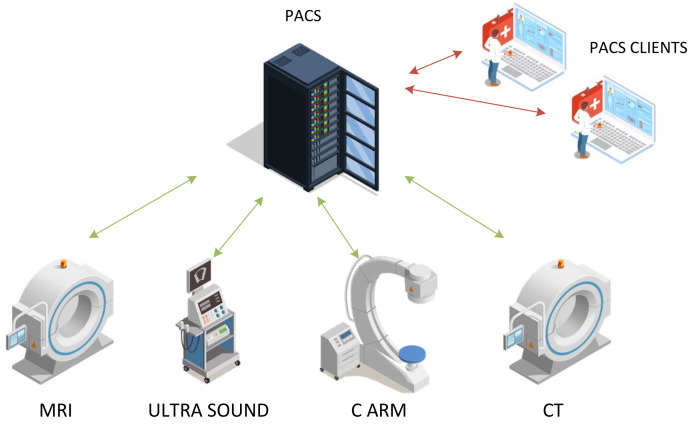
Picture Archiving and Communication System.

The DICOM committee regularly develops corrections and new chapters to the standard. Updates are compatible with previous versions, although some features may be abandoned in newer versions and the use of these features is not recommended in the new applications. The DICOM standard defines medical image exchange trough digital media (CD, DVD, etc.) and over the network, and it is designed to meet the needs of different imaging modalities (US, CT, MRI, etc.). DICOM is also designed to allow communication between different elements in the hospital medical imaging workflow usually organized as PACS, as shown in Figure 4.

The current version of the standard defines several transfer syntaxes for encapsulation of encoded pixel data including compressed data formats. DICOM includes Run Length Encoding (RLE), JPEG, JPEG-LS, JPEG-2000, JPIP, MPEG2, and MPEG-4AVC/H.264 [113]. On the other hand, the implementation of compression techniques in clinical practice is not an easy question. Although there is no reservation about the implementation of lossless compression methods, lossy compression has been an issue in the medical imaging community since the beginning.

In the United States, the Food and Drug Administration (FDA) regulates the use of medical devices. The FDA also regulates PACS performance such as medical image transmission, display, processing, and storage. In 1993, the FDA stated “the suitability of lossy compression for a variety of medical applications such as primary diagnosis, referral, and archiving”. In this guideline, manufacturers are not required to limit the use of PACS elements that use lossy compression but may limit their recommended use. These guidelines also stated that lossy compressed medical images should be marked and an approximate compression ratio should be obtained. The main concern is that degradation of image quality due to the lossy compression may result in a wrong diagnosis or patient treatment, and the manufacturer may be responsible for that [111]. This led to a more conservative approach in the commercial implementation of medical image compression, and lossless techniques become a technique of choice among DICOM recommended compression methods. It should be noticed that DICOM does not recommend or approve any particular compression for any particular modality or clinical application.

On the other hand, with the rapid development of sensors, communications, and image acquisition [37], compression of medical image data is critical, especially with limited bandwidth and data storage. In 2008, the Royal College of Radiologists (RCR) (UK), published a recommendation for lossy compression implementation in clinical contexts [114]. As part of the Connecting for Health national PACS solution, RCR recognizes the necessity of compression and recommends compression ratios for primary diagnosis for various modalities utilizing the JPEG2000 lossy compression approach. RCR recommendations are based on the review of earlier studies and they also recommend further studies for the assessment of the effects of compression on the thin slice for radiotherapy planning CT data. The Canadian Association of Radiologists (CAR) published a standard for lossy compression validation and identified examination categories in 2011 [115]. The maximum compression ratios for JPEG and JPEG2000 for certain modalities and anatomical locations are provided in this standard, which is based on Pan/Canadian studies [116]. To avoid apparent distortions for various examination types, the appropriate compression ratios were chosen based on a large-scale study with 100 readers. It is important to note that CAR clearly specifies that their research does not address image compression for CAD (Computer Aided Diagnosis) or image post-processing applications (3D reformatting, multiplanar reconstructions—MPR, maximum intensity projections—MIP, etc.). In 2009, the German Röntgen Society (DRG) organized a consensus conference with over 80 professionals to discuss the compression problem in digital medical imaging [117]. They looked at previous research and made suggestions for lossy JPEG and JPEG2000 compression ratios.

The ESR has launched an international expert discussion on the usability of lossy (irreversible) image compression in radiological imaging based on these findings and taking into account the major concerns of users and suppliers regarding the use of lossy image compression. In 2011, the discussion’s findings were published in a statement paper [118]. They define the term DAIC, assuming lossy compression that has no effect on a certain diagnostic task. In practice, this means that any compression-related artifacts should be invisible to the human eye or at such a low level that they have no effect on interpretation. Instead of making recommendations on appropriate compression ratios, the ESR suggests that “the radiologist should follow the CAR, DRG, or RCR recommendations to ensure diagnostically acceptable image compression (DAIC)”.

In 2017, ACR introduced a technical standard for the electronic practice of medical imaging [119]. In this standard, they do not propose “any general statement on the type or amount of compression that is appropriate to any particular modality, disease, or clinical application”, but advise responsible physicians to use scientific literature and national guidelines in deciding about the appropriate type of compression and appropriate compression ratio. They also stated that no matter if reversible or irreversible compression method is used, only algorithms defined by the DICOM standard such JPEG, JPEG-LS, JPEG-2000, or MPEG should be applied since images encoded with proprietary and nonstandard compression schemes reduce interoperability.

It has been demonstrated in recent analyses of medical image compression algorithms that techniques not included in the DICOM standard can provide a greater compression ratio [120]. It should also be mentioned that different compression ratios and approaches may be optimal for particular modalities or diagnostic tasks.

There are two primary issues with using irreversible compression techniques in medical imaging in general. Any irreversible compression method should first be tested and authorized by qualified physicians and/or professional organizations for a certain modality and exam, and then recognized and adopted into current standards. Because all applications and equipment are vendor-agnostic, the current position is convenient for both users and manufacturers. It is a significant achievement, and it is important to guarantee that it is maintained, especially in today’s connected world.

### 5.3. Future Medical Practices and Paradigms

Aging populations in many countries across the world are staining clinical institutions while healthcare costs are rising. This issue became more challenging during the pandemic when such clinical institutions were turned into COVID-19 treatment centers or they had to stop accepting patients when cases of COVID-19 were detected. According to [121], services such as artificial intelligence enabled remote patient monitoring and diagnoses can be supported by 5G and beyond 5G networks. Such networks can be employed to reliably connect hospitals, ambulances, and homes, thus making healthcare service more resource-efficient and more effective in managing resources and containing the outbreak of a pandemic. Authors in [122] have stated that ambulances featuring 5G connectivity can have high quality video calls with doctors and specialists in the hospital to attend the patient remotely especially under ongoing social distancing measures. Services such as AI-enabled remote patient monitoring and diagnoses can be supported by 5G networks and can be a useful life-saving solution for people living in remote areas. As stated in [121], travel restrictions in a pandemic can prevent patients and surgeons to attend more equipped hospitals. Given that surgeries and treatments must be offered all year round under any circumstances, 5G-enabled remote surgery, or in a larger scale internet of skills [123], enables the patients to attend a nearby hospital and receive treatment/surgery from a doctor hundreds of miles away. This of course will require Ultra-Reliable Low-Latency Communications (URLLC) and very high data-rate communications [124], which, for this work, are out of scope given that we are talking about IoT type of connectivity with limited capacity and data-rates. The technology presented in this paper may be more applicable on Predictive, Preventing and Personalized Medicine (PPPM). In the context of predictive and precision medicine, biomedical imaging techniques described in the previous two sections, are of particular importance. Moreover, the PPPM may be the key in health care for detecting and treating diseases early enough. With the advances in electronics, information communication technologies, and AI, it is now possible to consider employing PPPM to achieve effective and targeted preventive measures and to offer personalized treatment algorithms tailored to every individual. With the introduction of advanced healthcare, delayed interventional medicine may be replaced with predictive medicine and, therefore, treatment may also change from being reactive to preventive. Common non-communicable diseases such as diabetes, cardiovascular diseases, chronic respiratory diseases, cancer, and dental pathologies can be effectively treated or even prevented in some cases by introducing PPPM into our existing medical practices. With advanced healthcare, we can therefore introduce predictive diagnostics followed by targeted prevention before the manifestation of pathology.

## 6. Conclusions

Remote healthcare applications are needed more than ever, especially nowadays with the pandemic and lockdown restrictions where many people are unable to visit their local doctor and hospital for their routine checkups. Furthermore, hospitals cannot admit an increasing number of patients, and the healthcare system is required to provide its services at a home environment or temporary hospital. To support such services, we can make use of the current and future IoT technologies. This promising communication infrastructure may be used to support remote monitoring of patients and enhance virtual routine visits to doctors. The smart healthcare system should overcome many challenges.

Furthermore, large volumes of biomedical and healthcare data, in different formats and from heterogeneous sources, can be efficiently processed with advanced algorithms and technologies such as AI, ML, SDN, Cloud/Edge/Fog computing, etc. Due to the exponential growth of the IoT-enabled devices expected in the next ten years, medical devices will have to operate in a busy network environment of things. Reliability, resilience, and security are among the parameters that such networks must deliver. One solution is to optimize the transmission of these sensors by compressing their data and reducing their airtime. IoT architectures should be self-adaptive and energy-aware in order to provide efficient resource management and meet the requirements of next-generation healthcare applications. The diversity of these applications and their requirements makes the design process extremely complex. Therefore, the selection of communication standards, protocols, and technologies is a crucial step in providing the required performances. Secure mechanisms and protocols based on biometric and blockchain frameworks may open a new horizon in this emerging and challenging field.

In this paper, we discuss various emerging topics and challenges in the field of IoT for biomedical and healthcare systems. The future predictive, preventive, and personalized healthcare will strongly rely on emerging communication technologies, improved diagnostics from digital imaging and precision treatment, secure and reliable protocols, as well as optimized data transmission.

## Figures and Tables

**Table 1:** Data transmission requirements for wearable medical devices.

Sensor Type	Message Size [Byte]	Rate [msg/Day]
Glucose Sensor [21]	18	288
24H ECG Rec [22]	40	Real Time
Temperature [23]	8	6
Heart Rate [24]	4	6
Blood Pressure [25]	8	6
Blood Oxygen [25]	8	6

**Table 2:** Summary of data requirements and communication technologies for healthcare applications.

Sensors	Healthcare Application	IoT Communication Technology	Data Rate	Range	Advanced Technology	Benefits
Electrocardiography (ECG), Magnetocardiography (MCG)	Heart Activity Monitoring, Remote Health Monitoring, Arrhythmia Detection	Bluetooth/BLE Zigbee NB-IoT	3 Mbps/1 Mbps 20–250 kbps 200 kbps	10–100 m 100 m 1–10 km	AI-aided model for next-generation ultra-edge IoT sensors [82]	Remote patient monitoring for a prolonged period, especially in aging urban populations and under-served regions.
Heart Rate, Blood Pressure, Temperature	Heart Disease Detection, Remote Health Monitoring	Bluetooth/BLE LoRaWAN	3 Mbps/1 Mbps 50 kbps	10–100 m 2–20 km	Cloud-based heart disease prediction system using ML techniques [83]	Intelligent cloud-based network for analysis, planning and decision making. Provides continuous supervisions for patient’s safety.
Accelerometer, Gyroscope, Magnetometer	Fall Risk Prevention, Pervasive Healthcare Applications	Bluetooth/BLE EC-GSM-IoT	3 Mbps/1 Mbps 70–240 kbps	10–100 m 15 km	SDN-based multitier computing and communication architecture [84]	Real-time healthcare services. Performance advantages over traditional cloud-based approaches.
Blood Oxygen, Temperature, Heart Rate, Photoplethysmogram (PPG)	Remote Health Monitoring	Bluetooth/BLE LoRaWAN NB-IoT EC-GSM-IoT	3 Mbps/1 Mbps 50 kbps 200 kbps 70–240 kbps	10–100 m 2–20 km 1–10 km 15 km	Fog-based ML tools for data analysis and diagnosis [85]	Automated health monitoring and a COVID-safe framework that minimizes a coronavirus exposure risk. Smartphone application.

## Data Availability

Data from a healthy volunteer in Figure 3 are not publicly available due to the privacy constraints agreed upon within the signed informed consent.

## Abbreviations

The following abbreviations are used in this manuscript:

AAL	Ambient Assisted Living
ACR	American College of Radiology
AES	Advanced Encryption Standard
AI	Artificial Intelligence
API	Application Programming Interface
CE	Capsule Endoscopy
CT	Computed Tomography
GS	Glucose Sensors
BDA	Big Data Analytics
BLE	Bluetooth Low Energy
BP	Blood Pressure
BO	Blood Oxygen
CAR	Canadian Association of Radiologists
DAIC	Diagnostically Acceptable Irreversible Compression
DBPSK	Differential Binary Phase-Shift Keying
DICOM	Digital Imaging and Communication in Medicine
DSSS	Direct Sequence Spread Spectrum
EaaS	Entropy-as-a-Service
EC-GSM-IoT	Extended Coverage GSM Internet of Things
ECG	Electrocardiogram
EMG	Electromyography
ESI	Emergency Safety Index
ESR	European Society of Radiology
FDA	Food and Drug Administration
GFSK	Gaussian Frequency Shift Keying
GSM	Global System for Mobile communications
HR	Heart Rate
ICU	Intensive Care Unit
ISM	Industrial, Scientific and Medical
IoT	Internet of Things

LoRaWAN	Long Range Wider Area Network
LR-WPAN	Low Rate Wireless Personal Area Network
LP-WAN	Low Power Wide Area Network
LTE	Long Term Evolution
LTE CAT-N	Long Term Evolution Category N
LTE CAT-M	Long Term Evolution-Machine Type Communications
ML	Machine Learning
M2M	Machine to Machine
NB-IoT	Narrow Band Internet of Things
NFC	Near Field Communication
NB-IoT	Narrow Band Internet of Things
NEMA	National Electrical Manufacturers Association
PACS	Picture Archiving and Communication System
PHD	Personal Health Dashboard
PPG	Photoplethysmography
PPPM	Predictive, Preventing and Personalised Medicine
RCR	Royal College of Radiologists
RPMA	Random Phase Multiple Access
QoS	Quality of Service
SDN	Software Defined Networking
WBAN	Wireless Body Area Network
WPAN	Wireless Personal Area Network
WSN	Wireless Sensor Networks
MRI	Magnetic Resonance Imaging
URLLC	Ultra-Reliable Low-Latency Communications
US	Ultrasound
UWB	Ultra-Wideband
